# Ability of preoperative falls to predict postsurgical outcomes in non-selected patients undergoing elective surgery at an academic medical centre: protocol for a prospective cohort study

**DOI:** 10.1136/bmjopen-2016-011570

**Published:** 2016-09-21

**Authors:** Vanessa L Kronzer, Arbi Ben Abdallah, Sherry L McKinnon, Troy S Wildes, Michael S Avidan

**Affiliations:** Department of Anesthesiology, Washington University School of Medicine, St. Louis, Missouri, USA

**Keywords:** falls, preoperative, postoperative, patient-reported outcomes, functional dependence, quality of life

## Abstract

**Introduction:**

Falls are increasingly recognised for their ability to herald impending health decline. Despite the likely susceptibility of postsurgical patients to falls, a detailed description of postoperative falls in an unselected surgical population has never been performed. One study suggests that preoperative falls may forecast postoperative complications. However, a larger study with non-selected surgical patients and patient-centred outcomes is needed to provide the generalisability and justification necessary to implement preoperative falls assessment into routine clinical practice. The aims of this study are therefore twofold. First, we aim to describe the main features of postoperative falls in a population of unselected surgical patients. Second, we aim to test the hypothesis that a history of falls in the 6 months prior to surgery predicts postoperative falls, poor quality of life, functional dependence, complications and readmission.

**Methods and analysis:**

To achieve these goals, we study adult patients who underwent elective surgery at our academic medical centre and were recruited to participate in a prospective, survey-based cohort study called Systematic Assessment and Targeted Improvement of Services Following Yearlong Surgical Outcomes Surveys (SATISFY-SOS) (NCT02032030). Patients who reported falling in the 6 months prior to surgery will be considered ‘exposed.’ The primary outcome of interest is postoperative falls within 30 days of surgery. Secondary outcomes include postoperative functional dependence, quality of life (both physical and mental), in-hospital complications and readmission. Regression models will permit controlling for important confounders.

**Ethics and dissemination:**

The home institution's Institutional Review Board approved this study (IRB ID number 201505035). The authors will publish the findings, regardless of the results.

**Strengths and limitations of this study**

- Publishing protocols for observational studies is not routine practice, so this protocol sets a rigorous precedent for similar studies.
- This is the first trial to describe postoperative falls and the association between preoperative falls and postoperative falls, poor quality of life and functional dependence.
- The study population is both large (∼8000 patients) and non-selected.
- The enrolment rate of eligible patients is 65%, baseline survey response is 92% and 30-day survey response is 55%. Non-participation at any of these stages may introduce bias.
- Variables were not always available or derived from a validated tool.

## Introduction

This protocol followed published guidelines for protocols for observational studies, along with the Strengthening the Reporting of Observational Studies in Epidemiology (STROBE) checklist for cohort studies (see online supplementary material for the STROBE checklist).^1^ ^2^ For maximum transparency, we also added a limitations section. This protocol is V.01, written on 16 February 2016. The study will start after the protocol is accepted for publication (estimated study dates March 2016 to June 2016). See the Background section for literature review and justification for this study.

10.1136/bmjopen-2016-011570.supp1 Supplementary data

## Background

### Literature search and review

In preparation for this study, a systemised review of the literature was performed on preoperative and postoperative falls.^3^ This review showed that a limited number of studies on postoperative falls have been conducted, and the few that have been performed mostly study orthopaedic and elderly populations. While one study characterised postoperative falls in all surgery types, it was limited by its retrospective design, inpatient time period and exclusively male population.^4^ Another study suggested that most postoperative falls may be surgery related, especially in the first weeks following surgery. However, it only tracked falls that resulted in hospital care, and its follow-up period was limited to 90 days.^5^

Regarding the second aim of predicting postoperative outcomes, our systemised review identified just one paper that studied the association between preoperative falls and postoperative complications and readmission.^6^ However, the sample size was small (235), and the population included only men over 65 undergoing cardiac or colorectal surgery at a Veterans Affairs hospital. Using Medical Subject Heading (MeSH) terms of ‘accidental falls’, ‘postoperative period’ and each outcome, a thorough PubMed search was also performed to identify other relevant papers linking falls, surgery and each outcome of interest. This search showed that in non-surgical patients, falls are associated with greater functional dependence.^7^ ^8^ In addition, preoperative frailty and geriatric syndromes, which are linked to falls, are associated with postoperative complications^9–14^ and readmission.^15^ ^16^ Together, these findings suggest that falls before surgery may presage worse surgical outcomes. However, replication in a larger study is needed.

An additional literature search was performed to identify important confounders for each proposed outcome. Searches consisted of PubMed MeSH for each outcome, along with the MeSH, ‘Risk Factors’, in order to generate a thorough search. Variables that were strongly associated with the outcomes and that would be available to clinicians during preoperative assessment were included as confounders. In addition to confounders from the literature, our previous study on preoperative falls^17^ guided selection of confounders for postoperative falls. That is, variables with ORs of 1.5 or higher were included.

### Justification

This study is highly feasible since patients have been enrolling as part of the Systematic Assessment and Targeted Improvement of Services Following Yearlong Surgical Outcomes Surveys (SATISFY-SOS) study since July 2012.^18^ Questions about falls were added to the surveys on 29 October 2013. This information is currently available for abstraction.

As noted by our review article,^3^ postoperative falls are important yet have received relatively little attention. The review also concluded that many postoperative falls most likely occur beyond the hospitalisation period, and prospective design is most likely superior to retrospective design. Using both prospective design and an extensive follow-up period, this will be the first robust observational study to characterise postoperative falls in an unselected surgical population. Characterising features of postoperative falls such as their rate of occurrence, timing, injuries and risk factors will help raise awareness of this issue and assist clinicians in designing interventions to prevent them.

The preliminary research described above suggests that a history of preoperative falls may help predict adverse surgical outcomes. Nevertheless, the history of preoperative falls is not routinely assessed. Building on preliminary work in this area, this study offers replication and generalisability, which are needed to usher change into clinical practice. If our hypothesis is confirmed, preoperative falls will become an invaluable tool in improving preoperative decision-making and postoperative care.

## Specific aims

*Aim 1*: Describe characteristics of postoperative falls in an unselected surgical population, including their rate, timing, associated injuries and risk factors.

We hypothesise that the occurrence, injuries and risk factors of postoperative falls will be similar to those of preoperative falls, and the rate of postoperative falls will decline from 30-days after surgery to 1 year after surgery.

*Aim 2a*: Determine whether a preoperative history of falls serves as a useful tool for predicting falls in the 30 days (primary outcome) and 1 year following surgery of all types.

*Aim 2b*: Determine whether a preoperative history of falls predicts patient-centred outcomes including functionality and quality of life at 30 days and 1 year after surgery (secondary outcomes).

*Aim 2c*: Replicate findings that a preoperative history of falls predicts in-hospital surgical complications and readmission at 30 days (secondary outcomes).

We hypothesise that a history of preoperative falls will predict poor postsurgical outcomes, including postoperative falls, functional dependence, poor quality of life, in-hospital complications and readmission.

## Study design

A substudy of SATISFY-SOS, this prospective cohort study will synthesise three sources of information:

1. Preoperative ‘baseline’ survey—completed by patient at the preoperative assessment clinic within 4 weeks of surgery.
2. Postoperative ‘30-day’ and ‘1-year’ surveys—completed by patients ∼30 days and 1 year following surgery, respectively.
3. Electronic medical record—provides additional demographic and medical data.

The surveys were developed following a consultative process, including people with relevant expertise. The guiding principle was to formulate a survey that would be meaningful, reliable and practical for widespread dissemination to an unselected patient population. All surveys are available in the online supplementary material.

10.1136/bmjopen-2016-011570.supp2 Supplementary data

## Study groups

The target population for this study is patients who underwent preoperative assessment for elective surgery at Barnes Jewish Hospital in St. Louis, Missouri between 16 January 2014 and 7 October 2015. Over 70% of patients undergoing elective surgery are assessed by the centre for preoperative assessment and planning (CPAP) clinic before surgery. Reasons for no assessment include urgent surgery, geographical limitations or surgeon preference. Inclusion criteria include age 18 or older, ability to read the English consent form, ability to consent and plans to undergo elective surgery. During the analysis phase, patients are excluded from participation in SATISFY-SOS if they have undergone surgery within 60 days of their planned surgery date.

Approximately 65% of all eligible patients consent to participate in the study. Reasons for non-participation include patient refusal (∼70% of cases), lack of nurse time or training (∼20%), or lack of English literacy (∼10%). A study comparing participants to non-participants showed no major differences in characteristics.^19^ Approximately 92% of all consented patients complete a baseline survey. Reasons for lack of complete participation include insufficient time or changing their mind. Overall, the estimated number of eligible patients per YEAR is 15 500, the estimated number of consenting patients per year is 10 000, and the estimated number of baseline survey records per year is 9200. Approximately 16 000 baseline survey records are available in the time period specified for this study (16 January 2014 to 7 October 2015).

For the purposes of this substudy, only the first complete record for each patient (ie, baseline survey and corresponding 30-day survey) will be included in the final data set (∼94% of the available records). This practice ensures that each record is statistically independent from all the other records.

## Recruitment

Nurses at the CPAP clinic assess patient eligibility, recruit patients to participate, and obtain written consent using the consent form found in the online supplementary material. This consent form serves as the source of patient information for the study. While most patients decide whether or not to participate at this time, a patient can decide to participate any time between his or her CPAP visit and his or her surgery day. For patients who need special assistance, such as those who are blind or cannot physically sign a form, a witness can be obtained. However, in practice, this rarely occurs. No arrangements are made for non-English speakers, mentally ill, children, or those suffering from dementia since those are excluded groups (see the Study groups section). Patients receive no compensation for participation. If patients agree to participate, the CPAP nurse asks them to complete a brief baseline survey at the time of consent. Approximately 30 days and 1 year following surgery, they receive similar follow-up surveys. Both surveys were designed to take 10–15 min to complete. The SATISFY-SOS research team holds monthly update meetings with all CPAP nurses to inform them about study progress and to encourage optimal recruitment.

10.1136/bmjopen-2016-011570.supp3 Supplementary data

## Data

### Data to be collected

Incomplete follow-up is a major source of bias in many long-term observational cohort studies. To maximise completeness of 30-day and 1-year follow-up data, the following sequence of contact methods is performed. First, the survey is emailed up to three times starting ∼20 days after the surgery date. For those who did not provide an email or who do not respond to the emails, a paper survey is also mailed 25 days after the surgery date. If a patient does not respond to the first paper survey, a second paper survey is mailed 21 days later. If the second paper survey is not returned, the patient is phoned up to five times. Using this aggressive protocol, the 30-day survey response rate between 16 January 2014 and 7 May 2015 was ∼55%. An identical process occurs for the 1-year survey, beginning 365 days after the surgery date. For the date ranges included in this study, two versions of the 30-day survey (V.2 and V.3) and two versions of the 1-year survey (V.1 and V.2) were administered to participants. See online supplementary material to view these surveys.

10.1136/bmjopen-2016-011570.supp4 Supplementary data

10.1136/bmjopen-2016-011570.supp5 Supplementary data

10.1136/bmjopen-2016-011570.supp6 Supplementary data

10.1136/bmjopen-2016-011570.supp7 Supplementary data

Access to the SATISFY-SOS data is limited to investigators and staff who: have access to the Department of Anesthesiology desktop computers, have a computer account with the Department of Anesthesiology, use an IP address from within the department offices, have written permission to analyse the data from the principal investigator, and have authorised access to the database. For this project, the investigators formulated a list of requested data. They will provide that list to the informaticist, who manages the SATISFY-SOS database and has access to other medical records data. Using queries in MetaVision (iMDsoft, Needham, Massachusetts, USA), the informaticist will provide the requested survey and medical record data to the investigators. The query start date will be 16 January 2014 since important survey revisions occurred before this date.

Table 1 contains all variables to be collected for the study, along with the form of the variable, its source, time point for collection and whether or not it comes from a standardised tool. Of note, the informaticist performs rigorous data validation on each queried variable. If continuous variables are found to exhibit non-linearity, they will be categorised in accordance with their distribution.

**Table 1:** Characteristics of all variables used in this study

Type	Variable name	Form	Source	Time	Use of standardised tool
Explanatory	Preoperative falls	0, 1, 2, 3+	Survey	BL	Yes^21^
	Charlson Comorbidity Index	Ordinal, 0–12	CPAP H&P	BL	Yes^29^
	ASA physical status	Ordinal, 1–6	Anaesthesia record	Surgery	Yes^30^
	Procedural cardiac risk	Ordinal, 5 categories from high to low	CPAP clinician	BL	Yes^31^
Outcome	Postoperative falls	Binary (V.2); 0, 1, 2, 3+ (V.3)	Survey	30 days, 1 year	Yes^21^
	Functional dependence	Ordinal, 0–100 by 5	Survey	30 days, 1 year	Yes^32^
	Physical quality of life	Continuous, 0–100	Survey	30 days, 1 year	Yes^33^ ^34^
	Mental quality of life	Continuous, 0–100	Survey	30 days, 1 year	Yes^33^ ^34^
	In-hospital complications	Dichotomous	Survey	30 days	No
	Readmission	Dichotomous	Survey	30 days	No
Demographic	Age	Continuous	Medical record	BL	NA
	Sex	Dichotomous	Medical record	BL	NA
	Race	Nominal	Medical record (patient report)	BL	NA
	Ethnicity	Hispanic/Latino or not	Medical record (patient report)	BL	NA
	Body mass index	Continuous	CPAP nurse	BL	Yes
Confounder	Functional dependence	Ordinal, 0–100 by 5	CPAP nurse	BL	Yes^32^
	Physical quality of life	Continuous, 0–100	Survey	BL	Yes^33^ ^34^
	Mental quality of life	Continuous, 0–100	Survey	BL	Yes^33^ ^34^
	Smoking status	Current, past, never	CPAP physician or nurse	BL	NA
	Physical activity level	<4, 4–6, 6–10, >10 (METs)	CPAP physician	BL	Yes^35^
	Incontinence	Dichotomous (any issue)	CPAP nurse	BL	Yes^32^
	Toileting difficulty	Dichotomous (any issue)	CPAP nurse	BL	Yes^32^
	Mobility issue	Dichotomous (any issue)	CPAP nurse	BL	Yes^32^
	Neurological impairment (stroke, paraplegia or quadriplegia, Parkinson disease or multiple sclerosis)	Dichotomous	CPAP H&P	BL	No
	Chronic pain	Dichotomous	CPAP physician ROS	BL	No
	Dizziness/vertigo	Dichotomous	CPAP physician ROS	BL	No
	Depression	Dichotomous	CPAP H&P	BL	No
	Anxiety	Dichotomous	CPAP H&P	BL	No
Descriptive	Fall-related injury	Check all that apply	Survey	BL, 30 days, 1 year	No
	Days from BL to 30 days survey	Continuous	BL survey, Press Ganey	BL completion date to 30 days process date	NA
	Days from BL to 1 year survey	Continuous	BL survey, Press Ganey	BL completion date to 1 year process date	NA
	Surgery type	10 categories	Medical record	BL	NA
	In-hospital falls	Dichotomous	Survey (V.3 only)	30 days	Yes^21^
	In-hospital heart problem or stroke	Dichotomous	Survey	30 days	No
	In-hospital lung complication	Dichotomous	Survey	30 days	No
	In-hospital blood clot	Dichotomous	Survey	30 days	No
	In-hospital kidney or intestine complication	Dichotomous	Survey	30 days	No
	In-hospital nerve injury	Dichotomous	Survey	30 days	No
	In-hospital infection	Dichotomous	Survey	30 days	No
	In-hospital delirium	Dichotomous	Survey	30 days	No
	Other in-hospital complication	Dichotomous	Survey	30 days	No

ASA, American Society of Anesthesiology; BL, baseline; CPAP, centre for preoperative assessment and planning; H&P, history and physical; METs, metabolic equivalents; NA, not applicable; ROS, review of systems.

### Data handling and record keeping

All supplementary electronic data are collected from existing clinical records including MetaVision; CIDER; Compass; Allscripts TouchWorks; National Surgery Quality Improvement Program (NSQIP); and ClinDesk. SATISFY-SOS databases are hosted on a firewall-secured network server managed by the Department of Anesthesiology. The server is securely housed behind two locked doors within the departmental office suite and maintained and managed by the departmental IT team. The IT team patches and upgrades the server operating system on a routine basis and will employ virus protection and encryption to ensure proper data security. Incremental backups start daily; full backups start weekly. Only the project informaticist, data manager and director(s) have full access to these databases, which are also password-protected and encrypted for additional protection. Investigators have ‘read-only’ access to these databases for quality assessment and improvement.

Hard copies of the baseline surveys are collected daily from the CPAP clinic and securely stored behind two locked doors within the Department of Anesthesiology. Baseline completed paper surveys are scanned into a digital image format (compressed TIFF). The digital image files are indexed and stored on a research file server that is attached to a private network with no public access. Only Health Insurance Portability Accountability Act (HIPAA)-trained employees of the Department of Anesthesia or Barnes Jewish Healthcare are granted access to resources on this network. Access to the file server itself is further restricted to SATISFY-SOS team members.

Baseline surveys are processed by Solutions Data Systems. The digital image files are transmitted to Solutions Data Systems via secure file transfer protocol, which employs the same encryption method as secure web sites (128-bit or better Secure Sockets Layer (SSL)). Solutions Data Systems performs data entry of the digital image files, and prepares a monthly report of the data. That report is transmitted back to the research team via the same secure file transfer protocol connection. When data entry has been confirmed, Solutions Data Systems deletes the digital image file from its servers.

Press Ganey, a vendor specialising in patient survey distribution and collection disseminates, collects and processes 30-day and 1-year surveys. Its dedicated quality improvement team monitors and fine-tunes its quality assurance methods. For example, paper surveys processed through automated scanning are all manually checked, and a manager listens to 10% of telephone surveys.

Survey email, mail and call lists are generated at Washington University in a similar manner to mailing lists for billing services. For each patient and date of service, a unique ID is generated and never duplicated. This unique ID is a nonsensical number only meaningful to the research team. Initial survey collection attempts occur via email. The email letter contains a secure link to an encrypted electronic survey environment. This individual survey link can only be accessed once by the patient.

Patients without email addresses, or patients who did not respond to the email, are contacted by postal mail and then by telephone. On a weekly basis, packets specifically addressed to patients are generated. The packet includes a cover letter with the patient's name, mailing address and unique ID included. The cover letter is the only document with information linking the patient to a unique ID. The packet also includes a paper copy of the survey with only the patient's unique ID already printed on all pages as well as a stamped, self-addressed return envelope for return of the completed survey via the postal service. The survey pages will not include any protected health information. The survey responses are returned to a PO Box designated for this project.

Press Ganey picks up the surveys from the PO Box and converts them from a paper format to an electronic format with discrete variables, including the unique ID for each survey. Press Ganey makes image and data files available for File Transfer Protocol retrievals by the study team on a daily basis. For phone calls, Press Ganey uses an internal phone survey centre. All telephone surveys are recorded and available for future quality checks for performance improvement. These recordings are made available to the research team. The research team, at any time, may monitor real-time patient interviews for additional quality assurance purposes.

Press Ganey stores the survey hard copies for 90 days while the study team conducts spot check quality assessments of the scanned data. The company then shreds the paper copies. Similarly, Press Ganey will hold copies of the electronic files and electronic recordings for 90 days, after which the electronic files are removed permanently from its system (and then only maintained by Washington University). During this 90-day period, the study team conducts additional quality assessments of the converted data.

## Statistical considerations

### Sample size calculations

The required sample size is calculated based on the primary outcome, which is a fall within 30 days of surgery. Appropriate logistic regression models contain at least 10 ‘events’ per predictor variable.^20^ Since our prespecified model for postoperative falls includes 13 dependent variables (see the Analysis section), a sample with at least 130 events is required. In our population, preliminary work shows that ∼10% of patients report falling in the month following surgery. Thus, the minimum sample size needed for this study is 1300 patients.

Currently, our database contains over 9000 complete records (ie, baseline and 30-day survey) after the 16 January 2014 start date. To meet statistical assumptions of independent observations, we estimate that ∼600 records from patients with at least one previous entry in the data set will be excluded. We estimate that an additional 600 records will be excluded due to inappropriate time intervals (baseline survey to surgery <0 or >59 days; surgery to 30-day survey <20 or >120 days; or surgery to 1-year survey <275 or >455 days). Thus, our final sample size will be ∼7800.

Within this final sample, two versions of the 30-day (V.2 and V.3) and 1-year surveys (V.1 and V.2) were administered. Only V.3 of the 30-day survey and V.2 of the 1-year survey contain a validated falls question, as recommended by the Prevention of Falls Network Europe consensus group.^21^ The difference in question phrasing caused statistically significant differences in the percentage of patients reporting postoperative falls (5.5% vs 10% at 30 days and 18% vs 28% at 1-year, p<0.001). Out of the available records, about 20% (1500) completed V.3 of the 30-day survey, which is more than the 1300 required for the primary outcome. Thus, to maximise outcome validity, only these versions will be used for postoperative falls. In contrast, the readmission question contained only minor differences (‘Were you readmitted to the hospital?’ for V.2 vs ‘Check all that apply […] admitted to a hospital’ for V.3). The percentage reporting readmission was similar between surveys (5.2% vs 5.7%, p=0.416), so the results from both versions will be combined. The remaining outcomes were identical and will use all available data from both versions of the 30-day and 1-year surveys.

Comparing patients who reported falling preoperatively with those who did not, a sample size of 1500 has 80% power to detect a 58% increase in postoperative falls within 30 days. The estimated percentage of patients who experience functional decline in the month following surgery is 20%.^22^ Our own data suggest that the SD for quality of life is 11, 28% of patients experience in-hospital complications, and 5.5% of patients are readmitted within 30 days. Thus, a sample size of 7800 patients has 80% power to detect a 15% increase in functional decline after surgery, a 0.8 point difference in physical and mental qualities of life, a 12% increase in in-hospital complications, and a 34% increase in 30-day readmission. All of the above calculations used two tails, α=0.05 and a baseline fall proportion of 26%.^17^

The minimum important difference will be a 20% difference in proportions or ORs.^23^ From the calculations above, the detectable effect sizes for falls and readmission are larger than this prespecified threshold. However, the results of interest are the ORs from regression models rather than crude bivariate analysis. For quality of life estimates, the minimum important difference will be five points.^24^ ^25^

### Analysis

To begin analysis, we will perform descriptive statistics of all variables used in the analysis, using means, SDs and Student's t-tests for normal, continuous variables; medians, IQRs and Mann-Whitney tests for non-parametric, continuous variables; and proportions and χ^2^ tests for categorical variables. We will explore and report differences between patients with follow-up surveys at 30-days compared with non-responders.^26^ Among patients included in the analysis, we will also explore and report differences between those with complete records versus those with missing data. For variables with missing data, we will perform multiple imputation where appropriate.

Our first aim is to describe the characteristics of postoperative falls in an unselected surgical population, such as their occurrence, timing, injuries and risk factors. To achieve that goal, we plan to perform the following analyses:

- Descriptive statistics:
  Per cent who fell while still in the hospital;
  Number of falls (0, 1, 2, 3+) and fall rate in the 30 days and 1-year following surgery;
  Fall rate and per cent who fell by surgery type (10 surgery departments);
  Injuries from falls in the 30 days following surgery;
  Injuries from falls in the 1 year following surgery.
- ▸χ^2^:
  Proportion with any injury, comparing preoperative falls to postoperative falls at 30 days and 1 year;
  Proportion with severe injury, comparing preoperative falls to postoperative falls at 30 days and 1 year.
- Risk factors: see results from logistic regression model in Aims 2a–c.

As a subaim of Aim 1, we also plan to explore the rate of falls in the short-term versus long-term postoperative periods. To accomplish this, we will calculate the rate of falls per 100 person-years in the Behavioral Risk Factor Surveillance System (BRFSS) system, which serves as the control rate of falling in the general population of adults in the USA. We will then convert the 30-day, 6-month and 1-year fall rates from the SATISFY-SOS population into falls/(100 person-years) using the BRFSS distribution. Finally, we will compare the observed rate of falling to the general population's rate of falling at 1 month and 1 year (Student's t-test) to estimate how the rate of falls changes after surgery.

Aim 2 explores whether a preoperative history of falls serves as a useful tool for predicting surgical outcomes. First, we will calculate a crude dose–response relationship between the number of falls at baseline (0, 1, 2, 3+) and each outcome (postoperative falls, postoperative functional dependence, postoperative quality of life, in-hospital complications and 30-day readmission). For comparison, we will also calculate the crude dose–response relationship for a falls and injuries composite scale.^27^ We will examine the bivariate relationship between preoperative falls and each complication subcategory (heart/stroke, lung, blood clots, kidney/intestine, nerve injury, infection, delirium or other) using χ^2^ tests for the purpose of hypothesis generation.

To assess the relationship after controlling for important confounders, we will use logistic regression (categorical outcomes) and linear regression (continuous outcomes). Specifically, we will use forced entry of all prespecified variables. We will check all models for linearity of logit, multicollinearity, influential cases, conformity to linear gradients and goodness of fit. Since the number of possible interaction terms is large (between 45 and 91 per model), including all of them creates an unacceptably large risk of type I error. Therefore, our statistical consultant (Nan Lin) recommended selecting only 10% of the most clinically relevant interaction terms. Using a combination of prior research and clinical judgement, two investigators (VLK and MSA) selected the 10% most clinically relevant interaction terms for each model. The online supplementary material contains these prespecified interaction terms. Non-significant interaction terms (p>0.01) will be removed from the model.

10.1136/bmjopen-2016-011570.supp8 Supplementary data

Each model will include the following demographic and explanatory variables: age, sex, race (white vs non-white), BMI, preoperative falls history (0, 1, 2, 3+), Charlson Comorbidity Index (≥3 vs <3), American Society of Anesthesiology (ASA) physical status (≥3 vs <3) and procedural cardiac risk. On the basis of a careful literature search of each outcome variable (see the Literature search and review section), we selected confounding variables that are both routinely collected and known at the time of surgery planning. Outcome variables and their corresponding outcome-specific confounders are listed below.

- Presence or absence of at least one fall in the 30 days (primary outcome) and 1 year following surgery:
  Physical activity level (<4 vs >4 metabolic equivalents (METs));
  Baseline altered elimination (bowel or bladder);
  Baseline mobility abnormality;
  Dizziness/vertigo;
  Depression.
- ▸Barthel Index that is worse than baseline, at 30 days and 1 year after surgery:
  Baseline Barthel Index score;
  Neurological impairment;
  Mood disorder (depression or anxiety);
  Smoking status.
- ▸Physical quality of life score, at 30 days and 1 year after surgery:
  Baseline physical quality of life score;
  Preoperative physical activity level (<4 vs >4 METs);
  Chronic pain;
  Mood disorder (depression or anxiety);
  Smoking status.
- ▸Mental quality of life score, at 30 days and 1 year after surgery:
  Baseline mental quality of life score;
  Chronic pain;
  Mood disorder (depression or anxiety).
- ▸Presence or absence of any complication that occurred in the hospital, as reported in the 30-day survey:
  Smoking status.
- ▸Presence or absence of readmission at 30 days, as reported in the 30-day survey:
  In-hospital complications (30-day survey);
  Incontinence.

After running each model, we will substitute the number of falls (0, 1, 2, 3+) explanatory variable for the falls and fall injuries composite scale^27^ to evaluate which scale better predicts outcomes. Since we test multiple hypotheses, the threshold for significance will be α=0.01. We will report results using ORs and 99% CIs, along with variance estimates for regression parameters.

Using SAS/STAT software, V.9.4 (SAS Institute, Cary, North Carolina, USA), VLK will perform all analyses. Analysis will begin only after acceptance of this protocol for publication. This approach reassures readers that key analyses were truly prespecified rather than post hoc. If the authors decide to perform post hoc analyses, these will be described as such and will be performed for the purpose of further hypothesis generation.

## Limitations

This study contains several important limitations. First, the patients included in this study may represent a non-generalisable sample of the entire preoperative population. This study includes just one academic medical centre, and its patient population and rules for preoperative assessment clinic attendance may differ from that of other hospitals. In addition, only 65% of eligible preoperative assessment clinic attendees enrolled in the study, which may introduce bias. However, our analyses indicate that participants do not differ in important ways from non-participants. Furthermore, even if the sample was maximally biased, enrolling 65% of the actual target population means the results contain at least 80% accuracy.^28^ Another factor that biases the sample is non-response to the follow-up surveys. Although we mitigated non-response through an extensive follow-up protocol, only 55% of patients complete a 30-day survey.

The surveys also introduce several limitations. While the surveys are entitled ‘30-day’ and ‘1-year’ surveys, and most surveys occur near these times, the dates actually range from 20 to 120 and 275 to 455 days following surgery, respectively. In addition, each survey has two versions. Most questions are similar (readmissions) or identical (functional dependence, quality of life, complications) and can be combined. However, the postoperative falls question is significantly different. We will exclude results from the non-validated version, reducing the sample size available for this question. All important confounders will still be included, but the model may not have a sufficient number of outcomes to test all of the interactions of interest. Since many published papers do not test interactions, we do not think this compromises the robustness of the model.

The variables themselves also contain limitations. While the three explanatory variables and many of the outcome variables come from standardised tools, complications, readmission and fall-related injuries do not (see table 1). For complications and readmission, no gold standard currently exists. Thus, using patient-reported complications and readmission could be a weakness, or it could be a strength. Many variables (Charlson Index, neurological impairment, chronic pain, depression, anxiety) come from the CPAP history and physical or the CPAP physician review of systems, where the degree of completeness is unknown. To address this issue, the informaticist performs careful validation with cross-checks of patient records to ensure accuracy. For the procedural cardiac risk variable, patients do not always undergo the scored procedure. Finally, although the surveys, surgeon note, CPAP records and electronic medical record provide a large quantity of data, not all desired confounders identified through literature search are available. The confounders listed in this protocol are the ones that are available from our database.

## Compliance

### Subject compliance

Since the exposure for this study is patient report of falls within the 6 months before surgery, no procedures for monitoring exposure compliance are necessary. Methods to improve completion of 30-day and 1-year surveys are described in the Data to be collected section.

### Withdrawal of participants

Participants were withdrawn from the study only if requested. The reason for withdrawal is recorded by the team's clinical project specialist. The informaticist and Press Ganey are notified to ensure that the patient is no longer approached for data collection. As described in the consent form, data already collected may continue to be used.

## Ethical considerations

As a substudy of SATISFY-SOS, it has a waiver of informed consent. Written, informed consent is obtained from all participants for SATISFY-SOS (IRB ID number 201203088), and this consent form is included in the online supplementary material. As described in the Study groups section no special allowances are made for non-English speakers, children or mentally ill people. Participants may withdraw from the study at any time.

## Finance and insurance

Since this study is survey based, it involves no more than minimal risk to patients. Finance details, insurance details and cover for negligent and non-negligent harm are therefore not relevant.

## Reporting and dissemination

The results of this study will be presented at national meetings and published in a scientific journal. Participants will be individually notified of results only if discoveries are made that directly impact their health. The data and code for this project will be available on email request.
