# Supplementary material for: Ability of preoperative falls to predict postsurgical outcomes in non-selected patients undergoing elective surgery at an academic medical centre: protocol for a prospective cohort study
Source: BMJ Open. 2016 Sep 21;6(9):e011570. doi: 10.1136/bmjopen-2016-011570 (PMC5051422; doi:10.1136/bmjopen-2016-011570)
Supplement: Supplementary data [file bmjopen-2016-011570supp1.pdf]

STROBE Statement—checklist of items that should be included in reports of observational studies

	Item No	Recommendation
**Title and abstract**	1	(*a*) Indicate the study's design with a commonly used term in the title or the abstract<br>**DONE – Both title and abstract**
		(*b*) Provide in the abstract an informative and balanced summary of what was done and what was found.<br>**N/A – protocol only**
**Introduction**		
Background/rationale	2	Explain the scientific background and rationale for the investigation being reported<br>**DONE – See Background section**
Objectives	3	State specific objectives, including any prespecified hypotheses<br>**DONE – See Specific Aims section**
**Methods**		
Study design	4	Present key elements of study design early in the paper<br>**DONE – See Study Design section**
Setting	5	Describe the setting, locations, and relevant dates, including periods of recruitment, exposure, follow-up, and data collection<br>**DONE – See Study Groups section. Follow-up and data collection do not apply for protocols.**
Participants	6	*Cross-sectional study*—Give the eligibility criteria, and the sources and methods of selection of participants<br>**DONE – See Study Groups section.**
Variables	7	Clearly define all outcomes, exposures, predictors, potential confounders, and effect modifiers. Give diagnostic criteria, if applicable<br>**DONE – See Data section, except for effect modifiers, which are found in the supplementary material.**
Data sources/ measurement	8*	For each variable of interest, give sources of data and details of methods of assessment (measurement). Describe comparability of assessment methods if there is more than one group<br>**DONE – See Table 1**
Bias	9	Describe any efforts to address potential sources of bias<br>**DONE – see end of Recruitment section and beginning of Data to Be Collected section.**
Study size	10	Explain how the study size was arrived at<br>**DONE – see Sample size calculations section**
Quantitative variables	11	Explain how quantitative variables were handled in the analyses. If applicable, describe which groupings were chosen and why<br>**DONE – see Analysis section**
Statistical methods	12	Describe all statistical methods, including those used to control for confounding<br>**DONE – see Analysis section**
		(*b*) Describe any methods used to examine subgroups and interactions<br>**DONE – see Analysis section**
		(*c*) Explain how missing data were addressed<br>**DONE – see Analysis section**
		Cross-sectional study—If applicable, describe analytical methods taking account of

sampling strategy

DONE – see Analysis section

(*e*) Describe any sensitivity analyses

DONE – see Analysis section

Continued on next page

**Results**		
Participants	13*	Report numbers of individuals at each stage of study—eg numbers potentially eligible, examined for eligibility, confirmed eligible, included in the study, completing follow-up, and analysed<br>**DONE – see Study Groups section. Follow-up and analyzed portions do not apply as this is the study protocol.**
		(b) Give reasons for non-participation at each stage<br>**DONE – See Study Groups section.**
		(c) Consider use of a flow diagram<br>**Decided not to include since explanation is simple and straightforward.**
Descriptive data	14*	Give characteristics of study participants (eg demographic, clinical, social) and information on exposures and potential confounders<br>**N/A – protocol only**
		(b) Indicate number of participants with missing data for each variable of interest<br>**N/A – protocol only**
Outcome data	15*	
		Cross-sectional study—Report numbers of outcome events or summary measures<br>**N/A – protocol only**
Main results	16	(*a*) Give unadjusted estimates and, if applicable, confounder-adjusted estimates and their precision (eg, 95% confidence interval). Make clear which confounders were adjusted for and why they were included<br>**N/A – protocol only**
		(*b*) Report category boundaries when continuous variables were categorized<br>**N/A – protocol only**
Other analyses	17	Report other analyses done—eg analyses of subgroups and interactions, and sensitivity analyses<br>**N/A – protocol only**
**Discussion**		
Key results	18	Summarise key results with reference to study objectives<br>**N/A – protocol only**
Limitations	19	Discuss limitations of the study, taking into account sources of potential bias or imprecision. Discuss both direction and magnitude of any potential bias<br>**DONE – see Limitations section.**
Interpretation	20	Give a cautious overall interpretation of results considering objectives, limitations, multiplicity of analyses, results from similar studies, and other relevant evidence<br>**N/A – protocol only**
Generalisability	21	Discuss the generalisability (external validity) of the study results<br>**N/A – protocol only**
**Other information**		
Funding	22	Give the source of funding and the role of the funders for the present study and, if applicable, for the original study on which the present article is based<br>**DONE – see Funding section.**

*Give information separately for cases and controls in case-control studies and, if applicable, for exposed and unexposed groups in cohort and cross-sectional studies.

Note: An Explanation and Elaboration article discusses each checklist item and gives methodological background and published examples of transparent reporting. The STROBE checklist is best used in conjunction with this article (freely available on the Web sites of PLoS Medicine at http://www.plosmedicine.org/, Annals of Internal Medicine at http://www.annals.org/, and Epidemiology at http://www.epidem.com/). Information on the STROBE Initiative is available at www.strobe-statement.org.
