# Supplementary material for: Ability of preoperative falls to predict postsurgical outcomes in non-selected patients undergoing elective surgery at an academic medical centre: protocol for a prospective cohort study
Source: BMJ Open. 2016 Sep 21;6(9):e011570. doi: 10.1136/bmjopen-2016-011570 (PMC5051422; doi:10.1136/bmjopen-2016-011570)
Supplement: Supplementary data [file bmjopen-2016-011570supp2.pdf]

Please provide one answer for each question. Please circle your answer. If you are unsure how to answer a question, please choose the answer that fits best.

1. Why are you having this upcoming surgery? (Circle all that apply)
    - Treat or cure a medical condition
    - Decrease pain
    - Treat my symptoms
    - Improve my ability to perform daily life activities at home
    - Improve my abilities to perform daily life activities away from home
    - Improve my quality of life
    - Prolong my life
    - Doctor said I needed the surgery

2. Do you have pain now? (Check all that apply)
    - Yes, but NOT RELATED to my need for surgery
    - Yes, and RELATED to my need for surgery
    - No
    - Don't know

3. What is your expectation about pain a month after your surgery?
    - I have pain now and expect to have less pain after surgery
    - I have pain now and expect it to stay the same after surgery
    - I have pain now but I expect to have more pain after surgery
    - I have no pain now but I expect to have pain after surgery
    - I have no pain now and I do not expect to have pain after surgery
    - Don't know

4. In your opinion, how long would it take you to return to you normal life activities after you surgery:
    - Less than 1 week
    - 1-4 weeks
    - 1-3 months
    - 3-6 months
    - More than 6 months
    -

This section is about your general health. These questions do not necessarily relate to your upcoming procedure.

5. In the past six months, how many times have you had a fall, including a slip or trip in which you lost your balance and landed on the floor or ground or lower level?
    - Zero (0) (Please skip to question #6)
    - One time (1)
    - Two times (2)
    - Three or more (>2)

6. Did your fall result in any of the following? (Circle all that apply)
    - No injury
    - Bruising, sprains or cuts
    - Reduced mobility

- A fear of falling
- Severe pain
- Injury causing you to seek medical treatment
- Broken bone
- Head injury
- A change from independent living to assisted living

7. In general, would you say your health is:
    - Excellent
    - Very good
    - Good
    - Fair
    - Poor

8. Compared to one year ago, how would you rate your physical health in general now?
    - Much better
    - Slightly better
    - About the same
    - Slightly worse
    - Much worse

9. Compared to one year ago, how would you rate your emotional health now? (Such as feeling anxious, depressed or irritable)
    - Much better
    - Slightly better
    - About the same
    - Slightly worse
    - Much worse

10. Does your health now limit you in moderate activities, such as moving a table, pushing a vacuum cleaner, bowling, or playing golf? If so, how much?
    - Yes, limited a lot
    - Yes, limited a little
    - No, not limited at all

11. Does your health now limit you in climbing several flights of stairs? If so, how much?
    - Yes, limited a lot
    - Yes, limited a little
    - No, not limited at all

12. As a result of your physical health, during the past 4 weeks, have you accomplished less than you would like with your work or other regular daily activities?
    - No, none of the time
    - Yes, a little of the time
    - Yes, some of the time
    - Yes, most of the time
    - Yes, all of the time

13. As a result of your physical health, during the past 4 weeks, were you limited in the kind of work or other activities you can perform?
    - No, none of the time
    - Yes, a little of the time
    - Yes, some of the time
    - Yes, most of the time
    - Yes, all of the time

14. As a result of any emotional problems (such as feeling depressed or anxious), during the past 4 weeks, have you accomplished less than you would like with your work or other regular daily activities?
    - No, none of the time
    - Yes, a little of the time
    - Yes, some of the time
    - Yes, most of the time
    - Yes, all of the time

15. As a result of emotional problems (feeling depressed or anxious), during the past 4 weeks, have you not done work or other activities as carefully as usual?
    - No, none of the time
    - Yes, a little of the time
    - Yes, some of the time
    - Yes, most of the time
    - Yes, all of the time

16. During the past 4 weeks, how much did pain interfere with your normal work (including both work outside the home and housework)?
    - Not at all
    - A little bit
    - Moderately
    - Quite a bit
    - Extremely

17. How much of the time during the past 4 weeks have you felt calm and peaceful?
    - All of the time
    - Most of the time
    - A good bit of the time
    - Some of the time
    - A little bit of the time
    - None of the time

18. How much of the time during the past 4 weeks did you have a lot of energy?
    - All of the time
    - Most of the time
    - A good bit of the time
    - Some of the time
    - A little bit of the time

- None of the time

19. How much of the time during the past 4 weeks have you felt downhearted and blue?
    - All of the time
    - Most of the time
    - A good bit of the time
    - Some of the time
    - A little bit of the time
    - None of the time

20. How much of the time during the past 4 weeks has your physical health or emotional problems interfered with your social activities (like visiting with friends, relatives, etc.)?
    - All of the time
    - Most of the time
    - Some of the time
    - A little bit of the time
    - None of the time

21. If your ability to perform work is 10 when you are at your best and 0 when you are unable to work, circle the number that represents your ability to work this past week.
    0 1 2 3 4 5 6 7 8 9 10

22. What is your work status?
    - Employed
    - Volunteer work
    - Unemployed
    - Student
    - Homemaker
    - Retired (END OF SURVEY)
    - Disabled (END OF SURVEY)
23. Does your health limit you in your current job (work or studies or housework)?
    - I am not limited by my health
    - I am able to do my job with difficulty
    - I sometimes have to work slowly
    - I often have to work slowly
    - I am only able to do my job part time
    - I am entirely unable to do my job

24. Do you believe that, from the standpoint of your health, you will be able to do your current job in 1 year?
    - Yes
    - No
    - Not sure
