# Supplementary material for: Ability of preoperative falls to predict postsurgical outcomes in non-selected patients undergoing elective surgery at an academic medical centre: protocol for a prospective cohort study
Source: BMJ Open. 2016 Sep 21;6(9):e011570. doi: 10.1136/bmjopen-2016-011570 (PMC5051422; doi:10.1136/bmjopen-2016-011570)
Supplement: Supplementary data [file bmjopen-2016-011570supp4.pdf]

<u>The first section is about your specific health related to your recent surgery or procedure.</u> Please provide one answer for each question. Fill in the circle next to your answer. If you are unsure how to answer a question, please choose the one that fits best.

1. How would you rate your quality of life **now**?
- Better than before your procedure
- The same as before your procedure
- Worse than before your procedure
- Prefer not to answer

2. Since your procedure, have you been able to return to work?
- Yes
- No
- Does not apply
- Prefer not to answer

3. <u>While still in the hospital</u> after your recent procedure, did you have **PROBLEMS WITH YOUR HEART:** (Fill in all that apply)
- Heart attack?
- Your heart stopped beating (cardiac arrest)?
- Heart failure (congestive heart failure)?
- Abnormal heart rhythm such as atrial fibrillation?
- Severe pain coming from your heart (angina)?
- Prefer not to answer
- None

4. <u>While still in the hospital</u> after your recent procedure, did you have **PROBLEMS WITH BLOOD CLOTS**: (Fill in all that apply)
- Blood clot in your leg (Deep Vein Thrombosis)?
- Blood clot in your lung (Pulmonary Embolism)?
- Prefer not to answer
- None

5. <u>While still in the hospital</u> after your recent procedure, did you have **PROBLEMS WITH YOUR LUNGS & BREATHING**: (Fill in all that apply)
- You stopped breathing (respiratory arrest)?
- You were placed on a breathing machine because you were struggling to breathe on your own (respiratory failure)?
- An infection in your lungs (pneumonia)?
- Prefer not to answer
- None

6. <u>While still in the hospital</u> after your recent procedure, did you have **PROBLEMS WITH YOUR KIDNEYS OR INTESTINE**: (Fill in all that apply)
- Kidney failure and you needed kidney dialysis?
- GI bleed (internal bleeding from your stomach or intestine)?
- Stomach or intestinal ulcer?

- Prefer not to answer
- None

7. <u>While still in the hospital</u> after your recent procedure, did you have **PROBLEMS WITH PAIN OR NAUSEA**: (Fill in all that apply)

- Severe pain lasting for more than one day?
- Severe nausea and vomiting lasting for more than one day?
- Prefer not to answer
- None

8. <u>While still in the hospital</u> after your recent procedure, did you have **ANY OTHER PROBLEMS**: (Fill in all that apply)

- Delirium (temporary confusion with problems paying attention or thinking clearly)?
- Stroke (for example, weakness on one side of the body or difficulty speaking)?
- Nerve injury/paralysis related to your procedure?
- Infection in the surgical wound?
- Other (specify):____
- Prefer not to answer
- None

9. <u>After leaving the hospital</u>, did you need to seek medical treatment from a doctor's office, clinic or hospital?

- Yes
- No ***(skip to Question #18)***
- Prefer not to answer

10. Were you re-admitted into a hospital?

- Yes
- No
- Prefer not to answer

11. <u>After leaving the hospital</u>, did you seek medical treatment **FOR FOLLOW-UP**? (Fill in all that apply)

- You needed another procedure or follow-up from your surgery?
- On-going treatment such as chemotherapy or radiation?
- Prefer not to answer
- None

12. **<u>After leaving the hospital</u>**, did you seek medical treatment **FOR PROBLEMS WITH YOUR HEART**? (Fill in all that apply)

- Heart attack?
- Your heart stopped beating (cardiac arrest)?
- Heart failure (congestive heart failure)?
- Abnormal heart rhythm such as atrial fibrillation?
- Severe pain coming from your heart (angina)?
- Prefer not to answer
- None

13. **After leaving the hospital**, did you seek medical treatment **FOR PROBLEMS WITH BLOOD CLOTS**? (Fill in all that apply)

- Blood clot in your leg (Deep Vein Thrombosis)?
- Blood clot in your lung (Pulmonary Embolism)?
- Prefer not to answer
- None

14. **After leaving the hospital**, did you seek medical treatment **FOR PROBLEMS WITH YOUR LUNGS OR BREATHING**? (Fill in all that apply)

- You stopped breathing (respiratory arrest)?
- You were placed on a breathing machine because you were struggling to breathe on your own (respiratory failure)?
- An infection in your lungs (pneumonia)?
- Prefer not to answer
- None

15. **After leaving the hospital**, did you seek medical treatment **FOR PROBLEMS WITH YOUR KIDNEYS OR INTESTINE**? (Fill in all that apply)

- Kidney failure and you needed kidney dialysis?
- GI bleed (internal bleeding from your stomach or intestine)?
- Stomach or intestinal ulcer?
- Prefer not to answer
- None

16. **After leaving the hospital**, did you seek medical treatment **FOR PROBLEMS WITH PAIN OR NAUSEA**? (Fill in all that apply)

- Severe pain lasting for more than one day?
- Severe nausea and vomiting lasting for more than one day?
- Prefer not to answer
- None

17. **After leaving the hospital**, did you seek medical treatment **FOR ANY OTHER PROBLEMS**? (Fill in all that apply)

- Stroke (for example, weakness on one side of the body or difficulty speaking)?
- Nerve injury/paralysis related to your procedure?
- Infection in the surgical wound?
- Other (specify):____
- Prefer not to answer
- None

18. A fall is when your body goes to the ground without being pushed. **While still in the hospital or after leaving the hospital**, did you have a fall?

- Yes
- No
- Prefer not to answer

19. **While still in the hospital or after leaving the hospital**, have you had a problem with balance or walking?

- Yes
- No
- Prefer not to answer

20. **<u>After leaving the hospital,</u>** have you experienced any **Delirium** (temporary confusion with problems paying attention or thinking clearly)?

- Yes
- No
- Prefer not to answer

21. How does your CURRENT use of pain medications compare to your use BEFORE your procedure?

- I take LESS pain medication than before my procedure
- I take MORE pain medication than before my procedure
- I take the SAME amount of pain medication as I did before my procedure
- I take pain medications now, but did not before my procedure
- I am not taking pain medications now, and did not before my procedure
- Prefer not to answer

<u>This next section has to do with your anesthesia experience during your procedure</u>. Anesthesia is a combination of drugs or medicines used to either put patients to sleep or to sedate them to keep them from feeling pain during surgery and invasive medical procedures.

22. Did you have general anesthesia for your surgical procedure?

- Yes
- No ***(skip to Question #26)***
- I'm not sure
- Prefer not to answer

23. Do you remember anything in between going to sleep and waking up from your anesthesia?

- Yes
- No ***(skip to Question #26)***
- Prefer not to answer

24. Which of the following do you remember? (check all that apply)

- Hearing things?
- Feeling things (for example, the surgery or the sensation of the breathing tube in your throat)?
- Pain during your surgery?
- Feeling paralyzed during your surgery (feeling as if you could not move, speak or breathe)?
- Other: ____
- Prefer not to answer

25. Was this experience distressing to you?

- Yes
- No
- Prefer not to answer

The last few questions asked about a number of psychological symptoms. There are treatments available for these symptoms. Some of these treatments may be at no cost to you or at very low cost to you. If you would like to seek help for your symptoms, you can call the psychologist working on this study:
Dr. Thomas Rodebaugh, telephone: 314-935-8631.

<u>The next section is about your general health</u>. These questions <u>do not necessarily relate</u> to your recent procedure. Please provide one answer for each question. Fill in the circle next to your answer. If you are unsure how to answer a question, please choose the one that fits best.

- Excellent
- Very good
- Good
- Fair
- Poor
- Prefer not to answer

27. Does **your health now limit you** in **<u>moderate activities</u>**, such as moving a table, pushing a vacuum cleaner, bowling, or playing golf? If so, how much?

- Yes, limited a lot
- Yes, limited a little
- No, not limited at all
- Prefer not to answer

28. Does **your health now limit** you in climbing **<u>several</u>** flights of stairs? If so, how much?

- Yes, limited a lot
- Yes, limited a little
- No, not limited at all
- Prefer not to answer

29. As a result of your <u>physical health</u>, during the **past 4 weeks**, have you **<u>accomplished less</u>** than you would like with your work or other regular daily activities?

- No, none of the time
- Yes, a little of the time
- Yes, some of the time
- Yes, most of the time
- Yes, all of the time
- Prefer not to answer

30. As a result of your <u>physical health</u>, during the **past 4 weeks**, were you limited in the **<u>kind</u>** of work or other activities?

- No, none of the time
- Yes, a little of the time
- Yes, some of the time
- Yes, most of the time
- Yes, all of the time
- Prefer not to answer

31. As a result of any <u>emotional problems</u> (such as feeling depressed or anxious), during the **past 4 weeks**, have you **<u>accomplished less</u>** than you would like with your work or other regular daily activities?

- No, none of the time
- Yes, a little of the time
- Yes, some of the time

- Yes, most of the time
- Yes, all of the time
- Prefer not to answer

32. As a result of any emotional problems (such as feeling depressed or anxious), during the **past 4 weeks**, have you not done work or other activities as **carefully** as usual?

- No, none of the time
- Yes, a little of the time
- Yes, some of the time
- Yes, most of the time
- Yes, all of the time
- Prefer not to answer

33. During the **past 4 weeks**, how much did **pain** interfere with your normal work (including both work outside the home and housework)?

- Not at all
- A little bit
- Moderately
- Quite a bit
- Extremely
- Prefer not to answer

34. How much of the time during the **past 4 weeks** have you felt **calm and peaceful**?

- All of the time
- Most of the time
- A good bit of the time
- Some of the time
- A little bit of the time
- None of the time
- Prefer not to answer

35. How much of the time during the **past 4 weeks** did you have **a lot of energy**?

- All of the time
- Most of the time
- A good bit of the time
- Some of the time
- A little bit of the time
- None of the time
- Prefer not to answer

36. How much of the time during the **past 4 weeks** have you felt **downhearted and blue**?

- All of the time
- Most of the time
- A good bit of the time
- Some of the time
- A little bit of the time
- None of the time
- Prefer not to answer

37. How much of the time during the **past 4 weeks** has your **physical health or emotional problems** interfered with your social activities (like visiting with friends, relatives, etc.)?

- All of the time
- Most of the time
- Some of the time
- A little bit of the time
- None of the time
- Prefer not to answer

38. Compared to one year ago, how would you rate your **physical health** in general **now**?
- Much better
- Slightly better
- About the same
- Slightly worse
- Much worse
- Prefer not to answer

39. Compared to one year ago, how would you rate your **emotional problems now**? (Such as feeling anxious, depressed or irritable)
- Much better
- Slightly better
- About the same
- Slightly worse
- Much worse
- Prefer not to answer

40. On a scale of zero to ten, with ten being the worst pain and zero being no pain, please fill in your current pain level **when resting**.

0	1	2	3	4	5	6	7	8	9	10	Prefer not to answer
☐	☐	☐	☐	☐	☐	☐	☐	☐	☐	☐	☐

41. On a scale of zero to ten, with ten being the worst pain and zero being no pain, please fill in your current pain level **when moving (sitting up, walking or moving arms and legs)**.

0	1	2	3	4	5	6	7	8	9	10	Prefer not to answer
☐	☐	☐	☐	☐	☐	☐	☐	☐	☐	☐	___

42. If you have pain **at rest or when moving**, where is your pain located? (Fill in all that apply).
- Head or neck
- Chest
- Abdomen (belly)
- Upper Back
- Lower Back
- Arms
- Legs
- No pain
- Prefer not to answer

43. In the past 7 days has your thinking has been slow?
- Never
- Rarely (Once)

- Sometimes (Two or three times)
- Often (About once a day)
- Very often (Several times a day)
- Prefer not to answer

44. In the past 7 days has it seemed like your brain was <u>not</u> working as well as usual?

- Never
- Rarely (Once)
- Sometimes (Two or three times)
- Often (About once a day)
- Very often (Several times a day)
- Prefer not to answer

45. In the past 7 days have you had to work harder than usual to keep track of what you were doing?

- Never
- Rarely (Once)
- Sometimes (Two or three times)
- Often (About once a day)
- Very often (Several times a day)
- Prefer not to answer

46. In the past 7 days have you had trouble shifting back and forth between different activities that require thinking?

- Never
- Rarely (Once)
- Sometimes (Two or three times)
- Often (About once a day)
- Very often (Several times a day)
- Prefer not to answer

47. In the past 7 days has your mind been as sharp as usual?

- Not at all
- A little bit
- Somewhat
- Quite a bit
- Very much
- Prefer not to answer

48. In the past 7 days has your memory been as good as usual?

- Not at all
- A little bit
- Somewhat
- Quite a bit
- Very much
- Prefer not to answer

49. In the past 7 days has your thinking been as fast as usual?
- Not at all
- A little bit
- Somewhat
- Quite a bit
- Very much
- Prefer not to answer

50. In the past 7 days have you been able to keep track of what you are doing, even if you are interrupted?
- Not at all
- A little bit
- Somewhat
- Quite a bit
- Very much
- Prefer not to answer

The following questions are about your ability to care for yourself independently now. To be dependent means you need help with the task. To be independent means you can complete the task without help.

51. In relation to feeding yourself, you are…
- unable
- needing some help (i.e. cutting, spreading butter)
- independent
- Prefer not to answer

52. In relation to bathing/showering, you are…
- dependent
- independent
- Prefer not to answer

53. In relation to grooming, you are…
- needing some help with personal care
- independent (i.e. brushing hair, brushing teeth, shaving)
- Prefer not to answer

54. In relation to dressing, you are…
- dependent
- needing some help, but can do about half unaided
- independent (including buttons, zips, laces, etc.)
- Prefer not to answer

55. In relation to your bowels (defecation), you are…
- incontinent/unable to control bowels (or need to be given enemas)
- having occasional accidents
- continent/able to control bowels
- Prefer not to answer

56. In relation to your bladder (urination), you are…
- incontinent/unable to control bladder (or catheterized and unable to manage alone)
- having occasional accidents
- continent/able to control bladder
- Prefer not to answer

57. In relation to using the toilet, you are…
- dependent
- needing some help, but can do some things alone
- independent (on and off the toilet, dressing, wiping)
- Prefer not to answer

58. In relation to transferring from a bed to a chair and back, you are…
- unable (no sitting balance)
- needing major help but are able to sit (one or two people physically helping)
- needing minor help (verbal encouragement or physical help)
- independent
- Prefer not to answer

59. In relation to your mobility (walking) on level surfaces, you are…
- immobile (unable to walk or move about) for less than 50 yards
- wheelchair independent, including corners, greater than 50 yards
- walking with the help of one person (either verbal encouragement or physical help) greater than 50 yards
- independent (with or without a cane or walker) greater than 50 yards
- Prefer not to answer

60. In relation to climbing a flight of stairs, you are…
- unable
- needing help (verbal encouragement, physical help, carrying aid)
- independent
- Prefer not to answer
