# Supplementary material for: Ability of preoperative falls to predict postsurgical outcomes in non-selected patients undergoing elective surgery at an academic medical centre: protocol for a prospective cohort study
Source: BMJ Open. 2016 Sep 21;6(9):e011570. doi: 10.1136/bmjopen-2016-011570 (PMC5051422; doi:10.1136/bmjopen-2016-011570)
Supplement: Supplementary data [file bmjopen-2016-011570supp5.pdf]

<u>The first section is about your specific health related to your recent surgery or procedure.</u> Please provide one answer for each question. Fill in the circle next to your answer. If you are unsure how to answer a question, please choose the one that fits best. You may skip questions you wish not to answer.

1. How would you rate your quality of life **now**?
- Better than before your procedure
- The same as before your procedure
- Worse than before your procedure

2. Since your surgery, have you been able to return to work?
- Yes
- No
- Does not apply

3. **<u>While still in the hospital</u>** after your recent surgery, did you have **PROBLEMS WITH YOUR HEART:** (Fill in all that apply)
- Heart attack?
- Your heart stopped beating (cardiac arrest)?
- Heart failure (congestive heart failure)?
- Abnormal heart rhythm such as atrial fibrillation?
- Severe pain coming from your heart (angina)?
- None

4. **<u>While still in the hospital</u>** after your recent surgery, did you have **PROBLEMS WITH BLOOD CLOTS**: (Fill in all that apply)
- Blood clot in your leg (Deep Vein Thrombosis)?
- Blood clot in your lung (Pulmonary Embolism)?
- None

5. **<u>While still in the hospital</u>** after your recent surgery, did you have **PROBLEMS WITH YOUR LUNGS & BREATHING**: (Fill in all that apply)
- You stopped breathing (respiratory arrest)?
- You were placed on a breathing machine because you were struggling to breathe on your own (respiratory failure)?
- An infection in your lungs (pneumonia)?
- None

6. **<u>While still in the hospital</u>** after your recent surgery, did you have **PROBLEMS WITH YOUR KIDNEYS OR INTESTINE**: (Fill in all that apply)
- Kidney failure and you needed kidney dialysis?
- GI bleed (internal bleeding from your stomach or intestine)?
- Stomach or intestinal ulcer?
- None

7. **<u>While still in the hospital</u>** after your recent surgery, did you suffer from severe pain that required treatment?
- yes

- No (Skip to question 9)

8. Were you satisfied with the treatment given to you for your severe pain?
- Yes
- No

9. **<u>While still in the hospital</u>** after your recent surgery, did you have **ANY OTHER PROBLEMS**: (Fill in all that apply)
- A fall, including a slip or trip in which you lost your balance and landed on the floor or ground or lower level.
- Delirium (temporary confusion with problems paying attention or thinking clearly)?
- Stroke (for example, weakness on one side of the body or difficulty speaking)?
- Nerve injury/paralysis related to your procedure?
- Infection in the surgical wound?
- Other (specify):____
- None

10. **<u>After leaving the hospital following your surgery</u>**, did you receive medical care in any of the following locations? (please check all that apply)
- Outpatient clinic visit
- Urgent care center visit
- Emergency room visit
- Admitted to a hospital
- Admitted to a long-term care hospital or inpatient rehabilitation facility
- Underwent another surgery in an operating room
- Other location
- I did not receive any care

11. **<u>After leaving the hospital following your surgery</u>**, did you receive medical care for any of the following reasons? (please check all that apply)
- Routine medical care RELATED to your surgery
- Routine medical care NOT RELATED to your surgery
- Non-routine medical care RELATED TO your surgery
- Non-routine medical care NOT RELATED to your surgery
- Radiation therapy or chemotherapy
- I received care for a different reason
- I did not receive any care

12. **<u>After leaving the hospital</u>**, did you receive medical treatment **FOR PROBLEMS WITH YOUR HEART**? (Fill in all that apply)
- Heart attack?
- Your heart stopped beating (cardiac arrest)?
- Heart failure (congestive heart failure)?
- Abnormal heart rhythm such as atrial fibrillation?
- Severe pain coming from your heart (angina)?

13. **<u>After leaving the hospital</u>**, did you seek medical treatment **FOR PROBLEMS WITH BLOOD CLOTS**? (Fill in all that apply)

- Blood clot in your leg (Deep Vein Thrombosis)?
- Blood clot in your lung (Pulmonary Embolism)?
- None

14. **<u>After leaving the hospital</u>**, did you receive medical treatment **FOR PROBLEMS WITH YOUR LUNGS OR BREATHING**? (Fill in all that apply)

- You stopped breathing (respiratory arrest)?
- You were placed on a breathing machine because you were struggling to breathe on your own (respiratory failure)?
- An infection in your lungs (pneumonia)?
- None

15. **<u>After leaving the hospital</u>**, did you receive medical treatment **FOR PROBLEMS WITH YOUR KIDNEYS OR INTESTINE**? (Fill in all that apply)

- Kidney failure and you needed kidney dialysis?
- GI bleed (internal bleeding from your stomach or intestine)?
- Stomach or intestinal ulcer?
- None

16. **<u>After leaving the hospital</u>**, did you receive medical treatment for severe pain?

- Yes
- No

17. **<u>After leaving the hospital</u>**, did you receive medical treatment **FOR ANY OTHER PROBLEMS**? (Fill in all that apply) Need a discussion about free text from PG to see if other items should be added here.

- Stroke (for example, weakness on one side of the body or difficulty speaking)?
- Nerve injury/paralysis related to your procedure?
- Infection in the surgical wound?
- Other (specify):____
- None

18. Since your surgery, how many times have you had a fall, including a slip or trip in which you lost your balance and landed on the floor or ground or lower level?

- Zero (0) (Please skip to question #20)
- One time (1)
- Two times (2)
- Three or more (>2)

19. Did your fall result in any of the following? (Circle all that apply)

- No injury
- Bruising, sprains or cuts
- Reduced mobility
- A fear of falling
- Severe pain
- Injury causing you to seek medical treatment

- Broken bone
- Head injury
- A change from independent living to assisted living

20. How does your CURRENT use of pain medications compare to your use BEFORE your surgery?
- I take LESS pain medication than before my procedure
- I take MORE pain medication than before my procedure
- I take the SAME amount of pain medication as I did before my procedure
- I take pain medications now, but did not before my procedure
- I am not taking pain medications now, and did not before my procedure

This next section has to do with your anesthesia experience during your procedure. Anesthesia is a combination of drugs or medicines used to either put patients to sleep or to sedate them to keep them from feeling pain during surgery and invasive medical procedures.

21. Did you have general anesthesia for your surgical procedure?
- Yes
- No ***(skip to Question # 23)***
- I'm not sure

22. Do you remember anything in between going to sleep and waking up from your anesthesia?
- Yes
- No ***(skip to Question # 23)***

23. Was this experience distressing to you?
- Yes
- No

The last few questions asked about a number of psychological symptoms. There are treatments available for these symptoms. Some of these treatments may be at no cost to you or at very low cost to you. If you would like to seek help for your symptoms, you can call the psychologist working on this study: Dr. Michael Avidan, telephone: 314-286-1768.

The next section is about your general health. These questions do not necessarily relate to your recent procedure. Please provide one answer for each question. Fill in the circle next to your answer. If you are unsure how to answer a question, please choose the one that fits best.

24. In general, would you say your health is:
- Excellent
- Very good
- Good
- Fair
- Poor

25. Does **your health now limit you** in **moderate activities**, such as moving a table, pushing a vacuum cleaner, bowling, or playing golf? If so, how much?

- Yes, limited a lot
- Yes, limited a little
- No, not limited at all

26. Does **your health now limit** you in climbing **several** flights of stairs? If so, how much?

- Yes, limited a lot
- Yes, limited a little
- No, not limited at all

27. As a result of your physical health, during the **past 4 weeks**, have you **accomplished less** than you would like with your work or other regular daily activities?

- No, none of the time
- Yes, a little of the time
- Yes, some of the time
- Yes, most of the time
- Yes, all of the time

28. As a result of your physical health, during the **past 4 weeks**, were you limited in the **kind** of work or other activities?

- No, none of the time
- Yes, a little of the time
- Yes, some of the time
- Yes, most of the time
- Yes, all of the time

29. As a result of any emotional problems (such as feeling depressed or anxious), during the **past 4 weeks**, have you **accomplished less** than you would like with your work or other regular daily activities?

- No, none of the time
- Yes, a little of the time
- Yes, some of the time
- Yes, most of the time
- Yes, all of the time

30. As a result of any emotional problems (such as feeling depressed or anxious), during the **past 4 weeks**, have you not done work or other activities as **carefully** as usual?

- No, none of the time
- Yes, a little of the time
- Yes, some of the time
- Yes, most of the time
- Yes, all of the time

31. During the **past 4 weeks**, how much did **pain** interfere with your normal work (including both work outside the home and housework)?

- Not at all
- A little bit
- Moderately

- Quite a bit
- Extremely

32. How much of the time during the **past 4 weeks** have you felt **calm and peaceful**?
- All of the time
- Most of the time
- A good bit of the time
- Some of the time
- A little bit of the time
- None of the time

33. How much of the time during the **past 4 weeks** did you have **a lot of energy**?
- All of the time
- Most of the time
- A good bit of the time
- Some of the time
- A little bit of the time
- None of the time

34. How much of the time during the **past 4 weeks** have you felt **downhearted and blue**?
- All of the time
- Most of the time
- A good bit of the time
- Some of the time
- A little bit of the time
- None of the time

35. How much of the time during the **past 4 weeks** has your **physical health or emotional problems** interfered with your social activities (like visiting with friends, relatives, etc.)?
- All of the time
- Most of the time
- Some of the time
- A little bit of the time
- None of the time

36. Compared to one year ago, how would you rate your **physical health** in general **now**?
- Much better
- Slightly better
- About the same
- Slightly worse
- Much worse

37. Compared to one year ago, how would you rate your **emotional problems now**? (Such as feeling anxious, depressed or irritable)
- Much better
- Slightly better

- About the same
- Slightly worse
- Much worse

38. Currently, do you have any pain in the surgical incision or area related to your surgery?

- Yes
- No (Skip to question 42 if your answer is no)

39. Did the pain start after surgery?

- Yes
- No

40. On a scale of zero to ten, with zero being no pain and ten being the worst pain, please fill in your average pain level **during the last week**.

0	1	2	3	4	5	6	7	8	9	10
☐	☐	☐	☐	☐	☐	☐	☐	☐	☐	☐

41. If you have pain in surgical area, do you any of the following symptoms (check all that apply)

- Numbness
- Decrease sensation to cold or touch
- Increased sensation to cold or touch

42. In the past 7 days has your thinking has been slow?

- Never
- Rarely (Once)
- Sometimes (Two or three times)
- Often (About once a day)
- Very often (Several times a day)

43. In the past 7 days has it seemed like your brain was not working as well as usual?

- Never
- Rarely (Once)
- Sometimes (Two or three times)
- Often (About once a day)
- Very often (Several times a day)

44. In the past 7 days have you had to work harder than usual to keep track of what you were doing?

- Never
- Rarely (Once)
- Sometimes (Two or three times)
- Often (About once a day)
- Very often (Several times a day)

45. In the past 7 days have you had trouble shifting back and forth between different activities that require thinking?

- Never

- Rarely (Once)
- Sometimes (Two or three times)
- Often (About once a day)
- Very often (Several times a day)

46. In the past 7 days has your mind been as sharp as usual?
- Not at all
- A little bit
- Somewhat
- Quite a bit
- Very much

47. In the past 7 days has your memory been as good as usual?
- Not at all
- A little bit
- Somewhat
- Quite a bit
- Very much

48. In the past 7 days has your thinking been as fast as usual?
- Not at all
- A little bit
- Somewhat
- Quite a bit
- Very much

49. In the past 7 days have you been able to keep track of what you are doing, even if you are interrupted?
- Not at all
- A little bit
- Somewhat
- Quite a bit
- Very much

The following questions are about your ability to care for yourself independently now. To be <u>dependent</u> means you need help with the task. To be <u>independent</u> means you can complete the task without help.

50. In relation to feeding yourself, you are…
- unable
- needing some help (i.e. cutting, spreading butter)
- independent

51. In relation to bathing/showering, you are…
- dependent
- independent

52. In relation to grooming, you are…

- needing some help with personal care
- independent (i.e. brushing hair, brushing teeth, shaving)

53. In relation to dressing, you are…
- dependent
- needing some help, but can do about half unaided
- independent (including buttons, zips, laces, etc.)

54. In relation to your bowels (defecation), you are…
- incontinent/unable to control bowels (or need to be given enemas)
- having occasional accidents
- continent/able to control bowels

55. In relation to your bladder (urination), you are…
- incontinent/unable to control bladder (or catheterized and unable to manage alone)
- having occasional accidents
- continent/able to control bladder

56. In relation to using the toilet, you are…
- dependent
- needing some help, but can do some things alone
- independent (on and off the toilet, dressing, wiping)

57. In relation to transferring from a bed to a chair and back, you are…
- unable (no sitting balance)
- needing major help but are able to sit (one or two people physically helping)
- needing minor help (verbal encouragement or physical help)
- independent

58. In relation to your mobility (walking) on level surfaces, you are…
- immobile (unable to walk or move about) for less than 50 yards
- wheelchair independent, including corners, greater than 50 yards
- walking with the help of one person (either verbal encouragement or physical help) greater than 50 yards
- independent (with or without a cane or walker) greater than 50 yards

59. In relation to climbing a flight of stairs, you are…
- unable
- needing help (verbal encouragement, physical help, carrying aid)
- independent
