# Supplementary material for: Ability of preoperative falls to predict postsurgical outcomes in non-selected patients undergoing elective surgery at an academic medical centre: protocol for a prospective cohort study
Source: BMJ Open. 2016 Sep 21;6(9):e011570. doi: 10.1136/bmjopen-2016-011570 (PMC5051422; doi:10.1136/bmjopen-2016-011570)
Supplement: Supplementary data [file bmjopen-2016-011570supp6.pdf]

<u>The first section is about your specific health related to your surgery or procedure from about 1 year ago</u>. Please provide one answer for each question. Fill in the circle next to your answer. If you are unsure how to answer a question, please choose the one that fits best.

1. Since your surgery or medical procedure (about one year ago), have you been able to return to work?
- Yes
- No ***(skip to Question #4)***
- Does not apply ***(skip to Question #4)***
- Prefer not to answer***(skip to Question #4)***

2. How much are you able to work now?
- The same as before your procedure
- More than before your procedure
- Less than before your procedure
- Prefer not to answer

3. How long did it take for you to return to work?
- Less than 1 month
- 1 to 3 months
- 3 to 6 months
- More than 6 months
- Prefer not to answer

4. <u>Since completing the previous Health & Well-being Survey (about 9 to 11 months ago),</u> did you seek medical treatment from a doctor's office, clinic or hospital?
- Yes
- No ***(skip to Question #12)***
- Prefer not to answer

5. <u>Since completing the previous survey</u>, were you ever admitted into a hospital?
- Yes
- No
- Prefer not to answer

6. <u>Since completing the previous survey</u>, did you seek medical treatment **FOR FOLLOW-UP**? (Fill in all that apply)
- You needed another procedure or follow-up from your surgery?
- On-going treatment such as chemotherapy or radiation?
- Prefer not to answer
- None

7. **<u>Since completing the previous survey</u>**, did you seek medical treatment **FOR PROBLEMS WITH YOUR HEART**? (Fill in all that apply)

- Heart attack?
- Your heart stopped beating (cardiac arrest)?
- Heart failure (congestive heart failure)?
- Abnormal heart rhythm such as atrial fibrillation?
- Severe pain coming from your heart (angina)?
- Prefer not to answer
- None

8. **Since completing the previous survey**, did you seek medical treatment **FOR PROBLEMS WITH BLOOD CLOTS**? (Fill in all that apply)

- Blood clot in your leg (Deep vein thrombosis)?
- Blood clot in your lung (Pulmonary embolism)?
- Prefer not to answer
- None

9. **Since completing the previous survey**, did you seek medical treatment **FOR PROBLEMS WITH YOUR LUNGS OR BREATHING**? (Fill in all that apply)

- You stopped breathing (respiratory arrest)?
- You were placed on a breathing machine because you were struggling to breathe on your own (respiratory failure)?
- An infection in your lungs (pneumonia)?
- Prefer not to answer
- None

10. **Since completing the previous survey**, did you seek medical treatment **FOR PROBLEMS WITH YOUR KIDNEYS, STOMACH OR INTESTINE**? (Fill in all that apply)

- Kidney failure and you needed kidney dialysis?
- GI bleed (internal bleeding from your stomach or intestine)?
- Stomach or intestinal ulcer?
- Prefer not to answer
- None

11. **Since completing the previous survey**, did you seek medical treatment **FOR ANY OTHER PROBLEMS**? (Fill in all that apply)

- Stroke (for example, weakness on one side of the body or difficulty speaking)?

- Nerve injury/paralysis related to your procedure?
- Other (specify):____
- Prefer not to answer
- None

12. A fall is when your body goes to the ground without being pushed. **Since completing the previous survey**, did you have a fall?

- Yes
- No
- Prefer not to answer

13. **Since completing the previous survey**, have you had a problem with balance or walking?

- Yes
- No
- Prefer not to answer

14. Since completing the previous survey, have you experienced any Delirium (temporary confusion with problems paying attention or thinking clearly)?

- Yes
- No
- Prefer not to answer

15. How does your **CURRENT** use of pain medications compare to your use ONE YEAR AGO?

- I take LESS pain medication than I did one year ago
- I take MORE pain medication than I did one year ago
- I take the SAME amount of pain medication than I did one year ago
- I take pain medications now, but did not one year ago
- I am not taking pain medications now, and did not one year ago
- Prefer not to answer

The next section is about your CURRENT general health. These questions do not necessarily relate to your procedure from about 1 year ago. Please provide one answer for each question. Fill in the circle next to your answer. If you are unsure how to answer a question, please choose the one that fits best.

16. In general, would you say your health is:

- Excellent
- Very good
- Good
- Fair
- Poor
- Prefer not to answer

17. Does **your health now limit you** in **moderate activities**, such as moving a table, pushing a vacuum cleaner, bowling, or playing golf? If so, how much?

- Yes, limited a lot

- Yes, limited a little
- No, not limited at all
- Prefer not to answer

18. Does **your health now limit** you in climbing **several** flights of stairs? If so, how much?

- Yes, limited a lot
- Yes, limited a little
- No, not limited at all
- Prefer not to answer

19. As a result of your physical health, during the **past 4 weeks**, have you **accomplished less** than you would like with your work or other regular daily activities?

- No, none of the time
- Yes, a little of the time
- Yes, some of the time
- Yes, most of the time
- Yes, all of the time
- Prefer not to answer

20. As a result of your physical health, during the **past 4 weeks**, were you limited in the **kind** of work or other activities?

- No, none of the time
- Yes, a little of the time
- Yes, some of the time
- Yes, most of the time
- Yes, all of the time
- Prefer not to answer

21. As a result of any emotional problems (such as feeling depressed or anxious), during the **past 4 weeks**, have you **accomplished less** than you would like with your work or other regular daily activities?

- No, none of the time
- Yes, a little of the time
- Yes, some of the time
- Yes, most of the time
- Yes, all of the time
- Prefer not to answer

22. As a result of any emotional problems (such as feeling depressed or anxious), during the **past 4 weeks**, have you not done work or other activities as **carefully** as usual?

- No, none of the time
- Yes, a little of the time
- Yes, some of the time
- Yes, most of the time
- Yes, all of the time

- Prefer not to answer

23. During the **past 4 weeks**, how much did **pain** interfere with your normal work (including both work outside the home and housework)?

- Not at all
- A little bit
- Moderately
- Quite a bit
- Extremely
- Prefer not to answer

24. How much of the time during the **past 4 weeks** have you felt **calm and peaceful**?

- All of the time
- Most of the time
- A good bit of the time
- Some of the time
- A little bit of the time
- None of the time
- Prefer not to answer

25. How much of the time during the **past 4 weeks** did you have **a lot of energy**?

- All of the time
- Most of the time
- A good bit of the time
- Some of the time
- A little bit of the time
- None of the time
- Prefer not to answer

26. How much of the time during the **past 4 weeks** have you felt **downhearted and blue**?

- All of the time
- Most of the time
- A good bit of the time
- Some of the time
- A little bit of the time
- None of the time
- Prefer not to answer

27. How much of the time during the **past 4 weeks** has your **physical health or emotional problems** interfered with your social activities (like visiting with friends, relatives, etc.)?

- All of the time
- Most of the time
- Some of the time
- A little bit of the time
- None of the time

- Prefer not to answer

28. Compared to two years ago, how would you rate your quality of life **now**?

- Better
- The same
- Worse
- Prefer not to answer

29. Compared to two years ago, how would you rate your **physical** health in general **now**?

- Much better
- Slightly better
- About the same
- Slightly worse
- Much worse
- Prefer not to answer

30. Compared to two years ago, how would you rate your **emotional** problems **now**? (Such as feeling anxious, depressed or irritable)

- Much better
- Slightly better
- About the same
- Slightly worse
- Much worse
- Prefer not to answer

31. On a scale of zero to ten, with ten being the worst pain and zero being no pain, please fill in your current pain level **when resting**.

0	1	2	3	4	5	6	7	8	9	10	Prefer not to answer
☐	☐	☐	☐	☐	☐	☐	☐	☐	☐	☐	☐

32. On a scale of zero to ten, with ten being the worst pain and zero being no pain, please fill in your current pain level **when moving (sitting up, walking or moving arms and legs)**.

0	1	2	3	4	5	6	7	8	9	10	Prefer not to answer
☐	☐	☐	☐	☐	☐	☐	☐	☐	☐	☐	☐

33. If you have pain **at rest or when moving**, where is your pain located? (Fill in all that apply).

- Head or neck
- Chest
- Abdomen (belly)
- Upper Back
- Lower Back
- Arms
- Legs

34. In the past 7 days has your thinking been slow?

- Never
- Rarely (Once)
- Sometimes (Two or three times)
- Often (About once a day)
- Very often (Several times a day)
- Prefer not to answer

35. In the past 7 days has it seemed like your brain was <u>not</u> working as well as usual?

- Never
- Rarely (Once)
- Sometimes (Two or three times)
- Often (About once a day)
- Very often (Several times a day)
- Prefer not to answer

36. In the past 7 days have you had to work harder than usual to keep track of what you were doing?

- Never
- Rarely (Once)
- Sometimes (Two or three times)
- Often (About once a day)
- Very often (Several times a day)
- Prefer not to answer

37. In the past 7 days have you had trouble shifting back and forth between different activities that require thinking?

- Never
- Rarely (Once)
- Sometimes (Two or three times)
- Often (About once a day)
- Very often (Several times a day)
- Prefer not to answer

38. In the past 7 days has your mind been as sharp as usual?

- Not at all
- A little bit
- Somewhat
- Quite a bit
- Very much
- Prefer not to answer

39. In the past 7 days has your memory been as good as usual?

- Not at all
- A little bit
- Somewhat
- Quite a bit

- Very much
- Prefer not to answer

40. In the past 7 days has your thinking been as fast as usual?
- Not at all
- A little bit
- Somewhat
- Quite a bit
- Very much
- Prefer not to answer

41. In the past 7 days have you been able to keep track of what you are doing, even if you are interrupted?
- Not at all
- A little bit
- Somewhat
- Quite a bit
- Very much
- Prefer not to answer

The following questions are about your ability to care for yourself independently now. To be dependent means you need help with a task. To be independent means you can complete a task without help.

42. In relation to feeding yourself, you are…
- unable
- needing some help (i.e. cutting, spreading butter)
- independent
- Prefer not to answer

43. In relation to bathing/showering, you are…
- dependent
- independent
- Prefer not to answer

44. In relation to grooming, you are…
- needing some help with personal care
- independent (i.e. brushing hair, brushing teeth, shaving)
- Prefer not to answer

45. In relation to dressing, you are…
- dependent
- needing some help, but can do about half unaided
- independent (including buttons, zips, laces, etc.)
- Prefer not to answer

46. In relation to your bowels (defecation), you are…
- incontinent/unable to control bowels (or need to be given enemas)

- having occasional accidents
- continent/able to control bowels
- Prefer not to answer

47. In relation to your bladder (urination), you are…
- incontinent/unable to control bladder (or catheterized and unable to manage alone)
- having occasional accidents
- continent/able to control bladder
- Prefer not to answer

48. In relation to using the toilet, you are…
- dependent
- needing some help, but can do some things alone
- independent (on and off the toilet, dressing, wiping)
- Prefer not to answer

49. In relation to transferring from a bed to a chair and back, you are…
- unable (no sitting balance)
- needing major help but are able to sit (one or two people physically helping)
- needing minor help (verbal encouragement or physical help)
- independent
- Prefer not to answer

50. In relation to your mobility (walking) on level surfaces, you are…
- immobile (unable to walk or move about) for less than 50 yards
- wheelchair independent, including corners, greater than 50 yards
- walking with the help of one person (either verbal encouragement or physical help) greater than 50 yards
- independent (with or without a cane or walker) greater than 50 yards
- Prefer not to answer

51. In relation to climbing a flight of stairs, you are…
- unable
- needing help (verbal encouragement, physical help, carrying aid)
- independent
- Prefer not to answer
