# Supplementary material for: Ability of preoperative falls to predict postsurgical outcomes in non-selected patients undergoing elective surgery at an academic medical centre: protocol for a prospective cohort study
Source: BMJ Open. 2016 Sep 21;6(9):e011570. doi: 10.1136/bmjopen-2016-011570 (PMC5051422; doi:10.1136/bmjopen-2016-011570)
Supplement: Supplementary data [file bmjopen-2016-011570supp7.pdf]

<u>The first section is about your specific health related to your surgery or procedure from about 1 year ago</u>. Please provide one answer for each question. Fill in the circle next to your answer. If you are unsure how to answer a question, please choose the one that fits best. You may skip questions you wish not to answer.

1. Since your surgery (about one year ago), have you been able to return to work?
- Yes
- No ***(skip to Question #4)***
- Does not apply ***(skip to Question #4)***

2. How much are you able to work now?
- The same as before your surgery
- More than before your surgery
- Less than before your surgery

3. How long did it take for you to return to work?
- Less than 1 month
- 1 to 3 months
- 3 to 6 months
- More than 6 months
- Prefer not to answer

4. How long did it take you to return to your normal life activities after your surgery:
- less than 1 week,
- 1-4 weeks
- 1-3 months
- 3-6 months
- More than 6 months
- I have yet fully recovered

5. **<u>Since completing the previous Health & Well-being Survey (about 9 to 11 months ago),</u>** did you receive medical care in any of the following locations? (please check all that apply)
- Outpatient clinic visit
- Urgent care center visit
- Emergency room visit
- Admitted to a hospital
- Admitted to a long-term care hospital or inpatient rehabilitation facility
- Underwent another surgery in an operating room
- Other location
- I did not receive any care

6. **<u>Since completing the previous survey</u>**, did you receive medical care for any of the following reasons? (please check all that apply)
- Routine medical care RELATED to your surgery
- Routine medical care NOT RELATED to your surgery
- Non-routine medical care RELATED TO your surgery
- Non-routine medical care NOT RELATED to your surgery
- Radiation therapy or chemotherapy
- I received care for a different reason
- I did not receive any care?

7. **<u>Since completing the previous survey</u>**, did you receive medical treatment **FOR FOLLOW-UP**? (Fill in all that apply)

- You needed another procedure or follow-up from your surgery?
- On-going treatment such as chemotherapy or radiation?
- None

8. **Since completing the previous survey**, did you receive medical treatment **FOR PROBLEMS WITH YOUR HEART**? (Fill in all that apply)
   - Heart attack?
   - Your heart stopped beating (cardiac arrest)?
   - Heart failure (congestive heart failure)?
   - Abnormal heart rhythm such as atrial fibrillation?
   - Severe pain coming from your heart (angina)?
   - None

9. **Since completing the previous survey**, did you receive medical treatment **FOR PROBLEMS WITH BLOOD CLOTS**? (Fill in all that apply)
   - Blood clot in your leg (Deep vein thrombosis)?
   - Blood clot in your lung (Pulmonary embolism)?
   - None

10. **Since completing the previous survey**, did you receive medical treatment **FOR PROBLEMS WITH YOUR LUNGS OR BREATHING**? (Fill in all that apply)
    - You stopped breathing (respiratory arrest)?
    - You were placed on a breathing machine because you were struggling to breathe on your own (respiratory failure)?
    - An infection in your lungs (pneumonia)?
    - None

11. **Since completing the previous survey**, did you receive medical treatment **FOR PROBLEMS WITH YOUR KIDNEYS, STOMACH OR INTESTINE**? (Fill in all that apply)
    - Kidney failure and you needed kidney dialysis?
    - GI bleed (internal bleeding from your stomach or intestine)?
    - Stomach or intestinal ulcer?
    - None

12. **Since completing the previous survey**, did you receive medical treatment **FOR ANY OTHER PROBLEMS**? (Fill in all that apply)
    - Stroke (for example, weakness on one side of the body or difficulty speaking)?
    - Nerve injury/paralysis related to your procedure?
    - Other (specify):____
    - None

13. Since completing the previous survey, how many times have you had a fall, including a slip or trip in which you lost your balance and landed on the floor or ground or lower level?
    - Zero (0) (Please skip to question #20)
    - One time (1)
    - Two times (2)
    - Three or more (>2)

14. Did your fall result in any of the following? (Circle all that apply)
    - No injury
    - Bruising, sprains or cuts
    - Reduced mobility
    - A fear of falling

- Severe pain
- Injury causing you to seek medical treatment
- Broken bone
- Head injury
- A change from independent living to assisted living

15. How does your **CURRENT** use of pain medications compare to your use ONE YEAR AGO?
- I take LESS pain medication than I did one year ago
- I take MORE pain medication than I did one year ago
- I take the SAME amount of pain medication than I did one year ago
- I take pain medications now, but did not one year ago
- I am not taking pain medications now, and did not one year ago
- Prefer not to answer

<u>The next section is about your CURRENT general health</u>. These questions <u>do not necessarily</u> relate to your procedure from about 1 year ago. Please provide one answer for each question. Fill in the circle next to your answer. If you are unsure how to answer a question, please choose the one that fits best.

16. In general, would you say your health is:
- Excellent
- Very good
- Good
- Fair
- Poor

17. Does **your health now limit you** in **<u>moderate activities</u>**, such as moving a table, pushing a vacuum cleaner, bowling, or playing golf? If so, how much?
- Yes, limited a lot
- Yes, limited a little
- No, not limited at all

18. Does **your health now limit** you in climbing **<u>several</u>** flights of stairs? If so, how much?
- Yes, limited a lot
- Yes, limited a little
- No, not limited at all

19. As a result of your <u>physical health</u>, during the **past 4 weeks**, have you **<u>accomplished less</u>** than you would like with your work or other regular daily activities?
- No, none of the time
- Yes, a little of the time
- Yes, some of the time
- Yes, most of the time
- Yes, all of the time

20. As a result of your <u>physical health</u>, during the **past 4 weeks**, were you limited in the **<u>kind</u>** of work or other activities?
- No, none of the time
- Yes, a little of the time
- Yes, some of the time
- Yes, most of the time
- Yes, all of the time

21. As a result of any emotional problems (such as feeling depressed or anxious), during the **past 4 weeks**, have you **accomplished less** than you would like with your work or other regular daily activities?
- No, none of the time
- Yes, a little of the time
- Yes, some of the time
- Yes, most of the time
- Yes, all of the time

22. As a result of any emotional problems (such as feeling depressed or anxious), during the **past 4 weeks**, have you not done work or other activities as **carefully** as usual?
- No, none of the time
- Yes, a little of the time
- Yes, some of the time
- Yes, most of the time
- Yes, all of the time

23. During the **past 4 weeks**, how much did **pain** interfere with your normal work (including both work outside the home and housework)?
- Not at all
- A little bit
- Moderately
- Quite a bit
- Extremely

24. How much of the time during the **past 4 weeks** have you felt **calm and peaceful**?
- All of the time
- Most of the time
- A good bit of the time
- Some of the time
- A little bit of the time
- None of the time

25. How much of the time during the **past 4 weeks** did you have **a lot of energy**?
- All of the time
- Most of the time
- A good bit of the time
- Some of the time
- A little bit of the time
- None of the time

26. How much of the time during the **past 4 weeks** have you felt **downhearted and blue**?
- All of the time
- Most of the time
- A good bit of the time
- Some of the time
- A little bit of the time
- None of the time

27. How much of the time during the **past 4 weeks** has your **physical health or emotional problems** interfered with your social activities (like visiting with friends, relatives, etc.)?
- All of the time

- Most of the time
- Some of the time
- A little bit of the time
- None of the time

28. Compared to one year ago, how would you rate your **physical** health in general **now**?
- Much better
- Slightly better
- About the same
- Slightly worse
- Much worse

29. Compared to one year ago, how would you rate your **emotional** problems **now**? (Such as feeling anxious, depressed or irritable)
- Much better
- Slightly better
- About the same
- Slightly worse
- Much worse

30. Currently, do you have any pain in your surgical incision or in the area related to your surgery?
- Yes
- No (Skip to question 34)

31. Did this pain start after the surgery?
- Yes
- No

32. On a scale of zero to ten, with zero being no pain and ten being the worst pain, please fill in your average level of this pain **during the last week**.

0	1	2	3	4	5	6	7	8	9	10
☐	☐	☐	☐	☐	☐	☐	☐	☐	☐	☐

33. If you have pain in the surgical area, do you experience any of the following in that area symptoms (check all that apply)
- Numbness
- Decrease sensation to cold or touch
- Increased sensation to cold or touch

34. **In the past 7 days** has your thinking been slow?
- Never
- Rarely (Once)
- Sometimes (Two or three times)
- Often (About once a day)
- Very often (Several times a day)

35. **In the past 7 days** has it seemed like your brain was not working as well as usual?
- Never
- Rarely (Once)
- Sometimes (Two or three times)
- Often (About once a day)
- Very often (Several times a day)

***36.* In the past 7 days** have you had to work harder than usual to keep track of what you were doing?
- Never
- Rarely (Once)
- Sometimes (Two or three times)
- Often (About once a day)
- Very often (Several times a day)

***37.* In the past 7 days** have you had trouble shifting back and forth between different activities that require thinking?
- Never
- Rarely (Once)
- Sometimes (Two or three times)
- Often (About once a day)
- Very often (Several times a day)

***38.* In the past 7 days** has your mind been as sharp as usual?
- Not at all
- A little bit
- Somewhat
- Quite a bit
- Very much

***39.* In the past 7** days has your memory been as good as usual?
- Not at all
- A little bit
- Somewhat
- Quite a bit
- Very much

***40.* In the past 7 days** has your thinking been as fast as usual?
- Not at all
- A little bit
- Somewhat
- Quite a bit
- Very much

***41.* In the past 7 days** have you been able to keep track of what you are doing, even if you are interrupted?
- Not at all
- A little bit
- Somewhat
- Quite a bit
- Very much

The following questions are about your ability to care for yourself independently now. To be dependent means you need help with a task. To be independent means you can complete a task without help.

42. In relation to feeding yourself, you are…
- unable
- needing some help (i.e. cutting, spreading butter)
- independent

43. In relation to bathing/showering, you are…

- dependent
- independent

44. In relation to grooming, you are…
- needing some help with personal care
- independent (i.e. brushing hair, brushing teeth, shaving)

45. In relation to dressing, you are…
- dependent
- needing some help, but can do about half unaided
- independent (including buttons, zips, laces, etc.)

46. In relation to your bowels (defecation), you are…
- incontinent/unable to control bowels (or need to be given enemas)
- having occasional accidents
- continent/able to control bowels

47. In relation to your bladder (urination), you are…
- incontinent/unable to control bladder (or catheterized and unable to manage alone)
- having occasional accidents
- continent/able to control bladder

48. In relation to using the toilet, you are…
- dependent
- needing some help, but can do some things alone
- independent (on and off the toilet, dressing, wiping)

49. In relation to transferring from a bed to a chair and back, you are…
- unable (no sitting balance)
- needing major help but are able to sit (one or two people physically helping)
- needing minor help (verbal encouragement or physical help)
- independent

50. In relation to your mobility (walking) on level surfaces, you are…
- immobile (unable to walk or move about) for less than 50 yards
- wheelchair independent, including corners, greater than 50 yards
- walking with the help of one person (either verbal encouragement or physical help) greater than 50 yards
- independent (with or without a cane or walker) greater than 50 yards

51. In relation to climbing a flight of stairs, you are…
- unable
- needing help (verbal encouragement, physical help, carrying aid)
- independent
