# Supplementary material for: Ability of preoperative falls to predict postsurgical outcomes in non-selected patients undergoing elective surgery at an academic medical centre: protocol for a prospective cohort study
Source: BMJ Open. 2016 Sep 21;6(9):e011570. doi: 10.1136/bmjopen-2016-011570 (PMC5051422; doi:10.1136/bmjopen-2016-011570)
Supplement: Supplementary data [file bmjopen-2016-011570supp8.pdf]

Pre-Specified Interaction Terms

- Presence or absence of at least one fall in the 30 days (primary outcome) and 1 year following surgery. Note: Interaction terms will be included in the following order until the model reaches 10 outcomes per predictor variable:
    1. preoperative falls and Charlson Index
    2. preoperative falls and ASA Physical Status
    3. Charlson Index and ASA Physical Status
    4. age and depression
    5. preoperative falls and cardiac risk
    6. preoperative falls and depression
    7. preoperative falls and elimination problem (bowel or bladder)
    8. BMI and physical activity
    9. preoperative falls and physical activity
- Barthel Index that is worse than baseline, at 30 days and 1 year after surgery.
    - preoperative falls and Charlson Index
    - preoperative falls and ASA physical status
    - Charlson Index and ASA physical status
    - preoperative falls and cardiac risk
    - age and mood disorder
    - preoperative falls and mood disorder
    - ASA physical status and mood disorder
    - Neurological impairment and mood disorder
- Physical quality of life score, at 30 days and 1 year after surgery.
    - preoperative falls and Charlson Index
    - preoperative falls and ASA physical status
    - Charlson Index and ASA physical status
    - preoperative falls and cardiac risk
    - ASA physical status and chronic pain
    - chronic pain and mood disorder
    - age and mood disorder
    - age and chronic pain
    - BMI and preoperative physical status
    - preoperative falls and mood disorder
- Mental quality of life score, at 30 days and 1 year after surgery.
    - preoperative falls and Charlson Index
    - preoperative falls and ASA physical status
    - Charlson Index and ASA physical status
    - preoperative falls and cardiac risk
    - chronic pain and mood disorder
    - age and mood disorder
    - age and chronic pain
    - preoperative falls and mood disorder
- Presence or absence of any complication that occurred in the hospital, as reported in the 30-day survey.
    - preoperative falls and Charlson Index
    - preoperative falls and ASA physical status

- Charlson Index and ASA physical status
    - preoperative falls and cardiac risk
    - smoking and cardiac risk
- Presence or absence of readmission at 30 days, as reported in the 30-day survey.
    - preoperative falls and Charlson Index
    - preoperative falls and ASA physical status
    - Charlson Index and ASA physical status
    - preoperative falls and cardiac risk
    - preoperative falls and in-hospital complications
    - age and in-hospital complications
